# A procedure to correct proxy-reported weight in the National Health Interview Survey, 1976–2002

**DOI:** 10.1186/1478-7954-7-2

**Published:** 2009-01-06

**Authors:** Eric N Reither, Rebecca L Utz

**Affiliations:** 1 Population Research Laboratory, Department of Sociology, Social Work, and Anthropology, Utah State University, Logan, UT, USA; 2 Department of Sociology, University of Utah, Salt Lake City, UT, USA

## Abstract

**Background:**

Data from the National Health Interview Survey (NHIS) show a larger-than-expected increase in mean BMI between 1996 and 1997. Proxy-reports of height and weight were discontinued as part of the 1997 NHIS redesign, suggesting that the sharp increase between 1996 and 1997 may be artifactual.

**Methods:**

We merged NHIS data from 1976–2002 into a single database consisting of approximately 1.7 million adults aged 18 and over. The analysis consisted of two parts: First, we estimated the magnitude of BMI differences by reporting status (i.e., self-reported versus proxy-reported height and weight). Second, we developed a procedure to correct biases in BMI introduced by reporting status.

**Results:**

Our analyses confirmed that proxy-reports of weight tended to be biased downward, with the degree of bias varying by race, sex, and other characteristics. We developed a correction procedure to minimize BMI underestimation associated with proxy-reporting, substantially reducing the larger-than-expected increase found in NHIS data between 1996 and 1997.

**Conclusion:**

It is imperative that researchers who use reported estimates of height and weight think carefully about flaws in their data and how existing correction procedures might fail to account for them. The development of this particular correction procedure represents an important step toward improving the quality of BMI estimates in a widely used source of epidemiologic data.

## Background

**Figure 1:**
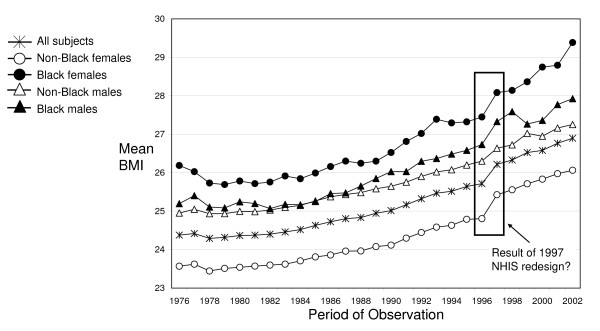
**Illustration of the larger-than-expected increase in BMI, NHIS 1976–2002**.

Trend data from the National Health Interview Survey (NHIS) illustrate how the U.S. population has gained weight steadily since the early 1980s (Figure 1). However, close inspection of NHIS data reveals an unusually rapid increase in body mass index (BMI = weight(kg)/height(m)^2^) between 1996 and 1997. This sudden increase is ubiquitous, although it is much less pronounced among non-Black males than in other race-sex groups.

In this study, we intend to show that the unusually large increase in mean BMI between 1996 and 1997 is primarily attributable to methodological changes in the NHIS. In 1997, the NHIS discontinued the practice of allowing proxy-reporting for adults [1,2], a practice where one adult could provide survey responses for other adults in the same household. Prior to the 1997 redesign, demographic and health information for adults in each household were collected through self-response interviews, as well as proxy-responses. The NHIS permitted two types of proxy-reporting for adults; (1) complete proxy-reported data, and (2) partial self-reports, which relied on a mixture of self-reports for some questions but proxy-reports for others.

Previous research has shown that proxy-reported height is a good indicator of self-reported height, but that proxy-reported weight tends to underestimate self-reported weight [3]. Thus, it seems probable that the elimination of proxy-reported height and weight in the 1997 NHIS caused mean BMI to increase suddenly in that year. While revised data collection procedures in the 1997 NHIS likely improved overall data quality, they also may have inadvertently contributed to a misleading impression about the pace of BMI increase in the U.S. population.

The objectives of this analysis were to explore the effect of proxy-reporting on population estimates of BMI and to develop a statistical correction which reduces the downward biases associated with proxy-reporting. Such a correction is imperative if researchers are to use NHIS data to monitor long-term changes in mean BMI and the prevalence of obesity in the U.S. population. Additionally, our study serves as a reminder about how data may be biased by routine data collection procedures. It provides an overview of potential reporting biases and offers statistical tools that may be employed to minimize such biases. These analyses are important for anyone using proxy-reported data and especially those interested in the validity of BMI measurements.

## Methods

### Study population

The NHIS is a repeated cross-sectional household survey of the noninstitutionalized civilian population in the U.S. [4] Its primary functions are to monitor the prevalence and distribution of disease and disability in the U.S. and assess patterns of health care utilization. Every week, interviewers from the U.S. Census Bureau conduct face-to-face interviews to gather information from "responsible family members" residing in randomly chosen households across the nation [5]. Households and the individuals within households are selected via a complex, multistage sampling design that involves both clustering and stratification. On average, Census personnel complete interviews at about 94% of the households selected.

This study merged NHIS data from 1976–2002 into a single database consisting of approximately 1.7 million adults aged 18 and over. Although the NHIS includes data on the health of children and adolescents, height and weight data from persons under 18 are not available. Thus, children were excluded from our sample. Although the NHIS began in 1957, it did not begin collecting data on weight and height until 1976. This timing is fortunate since available estimates suggest that the onset of the obesity epidemic occurred sometime in the 1980s [6].

The overarching motivation for the 1997 NHIS redesign was to streamline the questionnaire, improve its contents, and reduce the amount of time necessary to complete interviews, which had increased to an average of two hours by the mid-1990s [1]. Although sampling and interviewing procedures remained broadly intact, in 1997 the NHIS began to record survey responses with computer-assisted personal interviewing (CAPI) software on laptop computers, rather than the traditional paper and pencil method that had been used previously. Changes associated with the 1997 NHIS redesign have influenced the estimates for some conditions, such as asthma prevalence [2]. The 1997 redesign also affected estimates of BMI through, as we will show, the elimination of proxy-reporting.

### Measures

Body mass is measured with *body mass index *(BMI), calculated as weight(kg)/height(m)^2^. BMI is a widely used indicator of body mass because it controls for differences in weight due to height variations and has proven to be valid in population research [7,8]. Between 1976 and 1996, 5.5 percent of NHIS respondents had missing data on BMI; these cases were excluded from this analysis. (Note that some of our analyses extend only to 1996, as proxy-reporting was discontinued in 1997).

Reporting status was divided into three categories: *Self-report *designates persons who answered the entire survey for themselves. *Proxy-report *designates persons whose data were reported by another adult member of the household. Between 1976 and 1996, 477,703 individuals (about 31% of the NHIS sample) fit this description. *Partial self-report *designates persons whose data were a combination of self- and proxy-reports. This means that either the participant or another adult member of the household responded to questions regarding height and weight but, unfortunately, researchers cannot adjudicate between these two possibilities. Between 1976 and 1996, 81,405 participants (about 5% of the NHIS sample) were classified as partial self-reporters. Information on reporting status was unavailable for less than 1% of respondents from 1976–1996. Estimates of BMI for respondents with missing data on reporting status were, on average, comparable to self-reporters. Therefore, we excluded cases with missing reporting status.

Because both body mass and reporting status vary by race and sex, the sample was stratified into four race-sex groups: *Black Males*, *Black Females*, *Non-Black Males*, and *Non-Black Females*. A recent study using NHIS data found that differences between proxy- and self-reported health indicators narrowed substantially when respondent characteristics were taken into consideration [9]. Thus, our analyses controlled for basic sociodemographic variables that are known to be associated with body mass [10-14] and may also be associated with reporting status. *Period of observation *was grouped into four categories: 1976–84, 1985–88, 1989–92, and 1993–96. The initial category was broader than the others to capture enough partial self-reporters to produce stable parameter estimates. *Age *was grouped into six categories of approximately 10 years: 18–29, 30–39, 40–49, 50–59, 60–69, and 70 or older. *Marital status *was grouped into three categories: married with a spouse in the household, not currently married, and a category for unknown or missing data. The category "not currently married" included separated individuals and a very small proportion of married persons who indicated that their spouse was either absent or in an unknown location. Also, because the NHIS did not include "living with partner" as a response option until the 1997 redesign, we could not combine married with cohabiting individuals in our correction procedure. *Working status *was divided into three categories: working, not currently working, and a category for missing data. *Educational status *was grouped into five categories: less than a high school diploma, a high school diploma but no college experience, some college experience, a college degree or more, and a category for persons with missing data. Missing variable categories were necessary to maintain the full sample.

### Analytic plan

The analysis consisted of two parts: First, we estimated the magnitude of BMI differences by reporting status. Second, we developed a procedure to correct biases in BMI introduced by reporting status. This two-part analysis minimized the sudden increase in BMI that coincided with the 1997 NHIS redesign, resulting in more accurate trend estimates of mean BMI in the adult population from 1976–1996.

In part 1, we explored whether BMI differences by reporting status were the result of misreporting height, weight, or some combination of the two. (As discussed in more detail in the results section, analyses clearly showed that BMI differences were due to misreporting weight, not height). To verify that weight differences were caused by reporting status rather than other respondent characteristics (e.g., age and educational status), we evaluated parameter estimates in ordinary least square (OLS) regression models of weight on reporting status before and after the incorporation of a set of potential confounders. Although the parameter estimates from this part of the analysis could be used to correct differences in weight by reporting status, such an approach would assume that differences between proxy-, partial self-, and self-reports of weight were constant across sociodemographic strata.

Therefore, in part 2 we examined OLS regression models that incorporated interaction terms between reporting status and various respondent characteristics. Interaction terms causing model fit to improve significantly were included in the final correction procedure. Model fit was evaluated by a series of multiple partial *F *tests. This enabled us to determine whether the addition of *k *interaction terms $X_{1}^{*},...,X_{k}^{*}$ significantly improved the regression sum of squares beyond that contributed by the *p *variables *X*_1_,..., *X*_*p *_in the "full model" (i.e., *k *+ *p *variables), which consisted of all main effects and interaction terms [15]. That is,

$$F(X_{1}^{*},...,X_{k}^{*}|X_{1},...,X_{p})=\frac{[\sum_{i=1}^{n}{\left({\hat{Y}}_{i}-\bar{Y}\right)}^{2}full]-[\sum_{i=1}^{n}{\left({\hat{Y}}_{i}-\bar{Y}\right)}^{2}reduced]/k}{\left[\sum_{i=1}^{n}{\left(Y_{i}-{\hat{Y}}_{i}\right)}^{2}/Error\;df\;full\right]}$$

where ${\left[\sum_{i=1}^{n}({\hat{Y}}_{i}-\bar{Y}\right)}^{2}full]$ is the regression sum of squares in the full model consisting of all main effects and interaction terms, ${\left[\sum_{i=1}^{n}({\hat{Y}}_{i}-\bar{Y}\right)}^{2}reduced]$ is the regression sum of squares in the reduced model consisting of all main effects and interaction terms *except *for *k *interaction terms, and ${\left[\sum_{i=1}^{n}(Y_{i}-{\hat{Y}}_{i}\right)}^{2}/Error\;df\;full]$ is the mean square error in the full model.

We used SAS 9.1 to examine differences in body weight among self-, partial self-, and proxy-reporters [16]. We used Microsoft^® ^Excel in all graphics applications [17].

## Results

### Prevalence & accuracy of proxy-reporting

**Figure 2:**
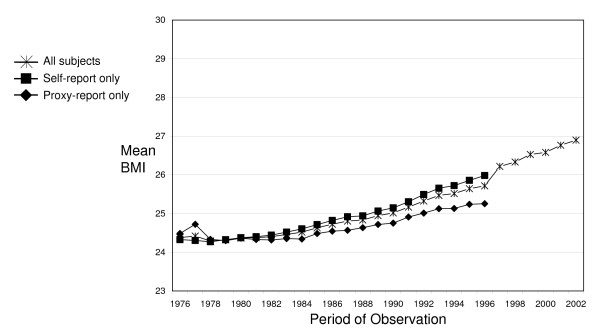
**Comparison of proxy- and self-reported BMI, NHIS 1976–2002**.

Between 1976 and 1996, around 50% of men responded to NHIS interviewers through either proxy- or partial self-reports. By contrast, women were more likely to respond via either proxy- or partial self-reports in later waves (20 percent in 1976 versus 30 percent in 1996). Figure 2 shows that mean BMI calculated from proxy-reported height and weight was generally lower than mean BMI calculated from self-reported height and weight. In fact, when only self-reported estimates of BMI are considered, the trend in mean BMI shows a smooth, consistent increase. This indicates that the elimination of proxy-reporting in 1997 is likely to be responsible for the sudden change in BMI estimates. Figure 2 also shows that while there was little difference between proxy- and self-reported data in the late 1970s, a difference emerged in the 1980s and grew steadily throughout the 1990s. By 1996, mean BMI for adults with proxy-reports (25.3) was well below mean BMI for self-reporters (26.0). This disparity varied across demographic categories; it was substantially larger for women than men and somewhat larger for Blacks than non-Blacks (stratified results available by request).

Measurements of height did not differ across self-report, proxy-report, or partial self- report data. For example, mean height among Black females was consistently reported at around 65 inches across the entire period of observation, regardless of reporting status. By contrast, there were substantial differences in mean weight by reporting status for all race-sex groups, except non-Black males (Table 1). Black males with self-report data were, on average, about 3 pounds heavier than Black males with proxy-report data. These differences were even more pronounced among females. Non-Black self-reporting females were, on average, about 6 pounds heavier than non-Black females with proxy-report data. Among Black females, this mean difference exceeded 10 pounds.

**Table 1:** Unstandardized coefficients in OLS regression models of weight (pounds) on reporting status and sociodemographic characteristics, NHIS 1976–1996

	Non-Black Males		Non-Black Females		Black Males		Black Females	
				
	Model 1	Model 2	Model 3	Model 4	Model 5	Model 6	Model 7	Model 8
*Intercept*	177.02**	159.81 **	142.26 **	136.49**	178.94**	161.01**	159.13 **	147.32**
*Reporting Status*								
Self-report	Referent	Referent	Referent	Referent	Referent	Referent	Referent	Referent
Partial self-report	1.16**	-0.84 **	-3.28 **	-3.83 **	-0.95 **	-1.82 **	-6.52 **	-5.13**
Proxy-report	0.37**	-1.27 **	-6.23 **	-5.31 **	-3.31 **	-3.43 **	-10.22 **	-7.15**
*Age*								
18–29		Referent		Referent		Referent		Referent
30–39		7.37 **		6.31**		6.84**		11.18**
40–49		10.88 **		10.86**		9.95**		18.59**
50–59		10.28 **		13.11**		10.52**		21.82**
60–69		6.99 **		11.38**		6.74**		16.79**
70 and older		-3.02 **		1.92**		-2.37**		3.11**
*Period*								
1976–1984		Referent		Referent		Referent		Referent
1985–1988		3.03 **		2.99**		2.78**		3.97**
1989–1992		5.01 **		5.15**		7.14**		7.46**
1993–1996		7.52 **		8.46**		11.04**		11.62**
*Marital Status*								
Not married		Referent		Referent		Referent		Referent
Married		6.66 **		0.10		7.52**		0.54*
Missing		4.81 **		-1.08*		2.87*		-2.06
*Working Status*								
Not working		Referent		Referent		Referent		Referent
Working		2.34 **		-0.55**		3.76**		-1.41**
Missing		0.93		-0.57		1.55*		0.74
*Educational Status*								
Less than high school		Referent		Referent		Referent		Referent
High school		4.44 **		-3.44**		2.03**		-4.35**
Some college		5.37 **		-5.34**		5.13**		-6.22**
College or more		2.27 **		-8.84**		4.82**		-10.88**
Missing		-2.64 **		-3.49**		-1.11*		-5.71**

* p < 0.05, ** p < 0.01

As expected, control variables were strong and statistically significant predictors of weight, although parameter estimates varied somewhat by race and sex (Table 1). For instance, married males weighed considerably more than non-married males, but little difference was observed between married and non-married females. More importantly, weight differences attributable to reporting status persisted after introducing potential confounders. Although accounting for differences in case-mix attenuated coefficients for partial self- and proxy-reported weight among Black and non-Black females, clear biases persisted after controlling for sociodemographic differences. Interestingly, the downward bias in proxy- and partial self-reported weight among non-Black males only emerged *after *the introduction of control variables. The introduction of control variables also caused the degree of bias to increase among Black males.

Multiple partial *F *ratios showed that reporting status interacted with blocks of sociodemographic variables, although the specific blocks of variables achieving statistical significance varied by race and sex (Table 2). For example, significant interactions were detected between proxy-reported weight and educational status among females of either race, but not males. Furthermore, within each race, a greater number of significant interactions were found among females. In the case of Black males, only marital status significantly interacted with either partial self- or proxy-reported weight.

**Table 2:** Multiple partial F-tests for blocks of interaction terms in OLS models of weight on reporting status and sociodemographic characteristics, NHIS 1976–1996

		Multiple Partial *F *Ratios			
		
	*k df*	Non-Black Males	Non-Black Females	Black Males	Black Females
*Partial Self-Report*					
by Age	5	2.14	2.60 *	0.19	1.98
by Period	3	10.22 **	8.75 **	1.13	1.47
by Marital Status	2	4.73 **	2.99	3.31 *	0.71
by Working Status	2	0.08	6.41 **	0.92	5.97 **
by Educational Status	4	1.35	2.03	1.91	0.35
*Proxy-Report*					
by Age	5	15.63 **	5.66 **	0.75	3.62 **
by Period	3	23.93 **	61.59 **	0.40	8.78 **
by Marital Status	2	56.95 **	27.22 **	19.48 **	1.19
by Working Status	2	4.77 **	25.64 **	0.59	16.97 **
by Educational Status	4	2.11	10.52 **	2.10	4.38 **

* p < 0.05, ** p < 0.01

### Correcting the bias associated with proxy-reporting

We estimated a final correction equation that included the main effects for reporting status, plus the significant blocks of interaction terms for each race-sex group:

*Adjusted weight *= *β*_0 _+ ((-1) * (*β*_1_X_1 _+ *β*_2_X_2 _+ *β*_(*i*...*n*)_X_1_X_(*i*...*n*) _+ *β*_(*j*...*n*)_X_2_X_(*j*...*n*)_)),

where *β*_0 _is the reported weight of the respondent, *β*_1 _is the main effect of partial self-reporting on weight, *β*_2 _is the main effect of proxy-reporting on weight, *β*_(*i*...*n*)_X_1_X_(*i*...*n*) _is the constellation of *i *to *n *interaction terms associated with partial self-reporting, and *β*_(*j*...*n*)_X_2_X_(*j*...*n*) _is the constellation of *j *to *n *interaction terms associated with proxy-reporting. Note that since X_1 _and X_2 _equal zero among self-reporters, all terms fall out of the equation except *β*_0_.

Incorporation of the interaction terms resulted in a more refined correction procedure that accounted for important sociodemographic differences in proxy- and partial self-reported estimates of weight. To illustrate, proxy estimates of weight among married non-Black males were, on average, about 2 pounds higher than proxy estimates of weight among non-Black males who were not currently married. Similarly, the downward bias in proxy-reported weight among non-Black females was about 3.5 pounds higher in 1993–96 than in the initial period of observation.

**Figure 3:**
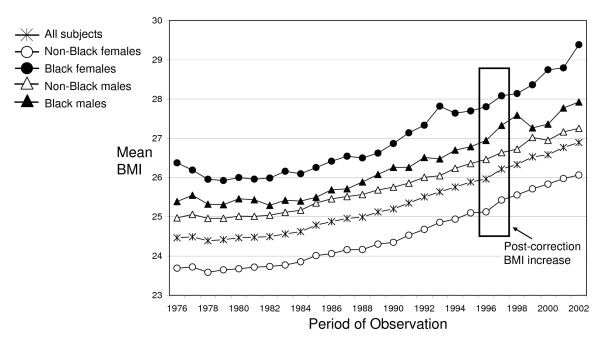
**Illustration of the increase in BMI after implementing a correction procedure, NHIS 1976–2002**.

Using the estimates of adjusted weight from the final correction equations for each race-sex group (parameter estimates available by request), we recalculated BMI for participants with proxy- or partial self-reported weight. Figure 3 shows the adjusted BMI trend for each race-sex group. When compared to Figure 1, Figure 3 illustrates that our correction procedure was remarkably effective at removing the larger-than-expected increase in BMI introduced at the time of the 1997 NHIS redesign. The sudden upward shift in mean BMI between 1996 and 1997 that was evident for most groups prior to adjustment largely disappears as a result of this correction procedure.

## Discussion

This study explored how BMI estimates in a large, nationally representative health survey varied depending on reporting status. Although not the focus of this analysis, extant research has found that self-reported BMI tends to underestimate clinical assessments of BMI [18-24]. This analysis focused specifically on the differences between proxy-reported and self-reported BMI, finding that BMI from proxy-reports was substantially lower than self-reported BMI. In other words, anthropometric data collected through proxy-reports introduced even more measurement error than self-reported data collection techniques. Consistent with our finding, research has shown that parents misestimate the height and weight of their preschool-aged children, producing downwardly biased estimates of BMI [25]. Therefore, relying on proxies such as spouses or parents to provide information about body mass introduces significant measurement error into an analysis and should be avoided whenever possible. However, because the NHIS and other large-scale epidemiologic studies have used proxy-report techniques to measure BMI (e.g., the National Long-Term Care Survey, the Ontario Familial Colon Cancer Registry, and the Continuing Surveys of Food Intakes by Individuals), and because these studies are commonly used to develop policy and to evaluate population health trends, it is imperative to understand and adjust for the measurement error associated with this type of data collection practice.

In summary, we found that the downward biases in BMI associated with proxy-reports were caused primarily by the underestimation of weight and that these underestimates varied systematically: First, the amount of misreporting differed by reporting status, with proxy-reports of weight showing more downward bias than partial self-reports. Second, misreporting differed by race, sex and other respondent characteristics. Third, the disparity between self- and proxy-reported weight increased substantially in recent waves of the NHIS. Given these patterns, we devised a correction procedure for each race-sex group that accounted for reporting status, age, period of observation, marital status, employment, and education. This correction procedure substantially reduced the larger-than-expected increase in BMI that coincided with the elimination of proxy-reporting in the 1997 NHIS redesign. Researchers interested in using results from this study to correct proxy-reported weight in the NHIS are encouraged to contact the authors for additional information.

The analyses presented here demonstrate that biases associated with proxy-reported weight have increased over the past few decades. Although the underestimation of weight appears particularly acute among proxy-reporters, the rise in obesity prevalence has presumably led to more widespread underreporting of weight by all NHIS respondents. If true, this would corroborate previous research showing that overweight subjects tend to underreport their weight to a greater extent than non-overweight subjects [23]. Given the likelihood of increasing biases in self-reported weight over time, future research should explore period trends in the underestimation of BMI in the NHIS. Presuming that research verifies that the downward bias in mean BMI has grown in recent years, a correction procedure should be devised so that NHIS data may be used to provide a more accurate assessment of trends associated with the U.S. obesity epidemic.

As noted, this study found significant differences in the amount of reporting bias among different demographic groups. For instance, females with proxy-reported estimates of BMI had greater measurement error than males with proxy-reported estimates. Also, proxy-reports for married males had less bias than proxy-reports for unmarried males. Assuming that a spouse is often the proxy respondent for a married participant, it appears that wives may report their husbands' weight more accurately than husbands report their wives' weight, and that the proxy respondent for an unmarried person may not know details such as height and weight as well as a spouse does. Our analyses also found that reporting biases were greatest among those from lower socioeconomic status groups (e.g., those with less than a high school education and those not currently working), suggesting that the validity of proxy-reports may be associated with cognitive traits influenced by socioeconomic attainment [26]. These demographic differences offer insight on which groups may provide more valid and reliable sources of proxy-report data. Should proxy-reports of weight be used in future study designs, it appears that females, particularly wives, and those with higher socioeconomic attainment provide more valid estimates than men or those of lower socioeconomic attainment.

**Figure 4:**
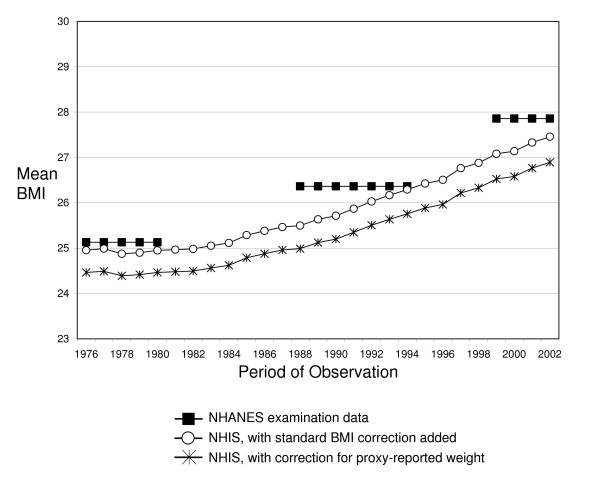
**Comparison of corrected NHIS estimates of BMI to NHANES examination data, 1976–2002**.

The correction procedure developed in this study accounted for the downward biases associated with proxy-reporting in the NHIS prior to 1997. However, as shown in Figure 4, our adjusted estimates underestimate clinically assessed measures of BMI from comparable time points from the National Health and Nutrition Examination Survey (NHANES). In fact, less than optimal results were produced even when we combined our correction for proxy-reported weight with a widely used BMI correction procedure that adjusts self-reported estimates to approximate clinically-assessed measurements [18]. Although the correspondence between corrected NHIS estimates of BMI and NHANES examination data is reasonably good from 1976–1980, it deteriorates substantially thereafter. Preliminary analyses suggest two reasons for this: First, self-reported estimates of BMI in NHIS are consistently lower than self-reported estimates of BMI in NHANES, and it is the latter estimates that have been used to develop BMI correction procedures. Second, the downward bias in NHIS estimates of BMI appears to have increased in recent years, which is consistent with the observation that underreporting of weight is most common among overweight individuals [23].

The discrepancy between corrected NHIS estimates of BMI and NHANES examination data reveals an important limitation of our study. However, this limitation also points to an opportunity for future research to build upon our study by developing a BMI correction for NHIS data that, in addition to biases in proxy-reporting, accounts for other shortcomings of NHIS data, such as increasing downward biases in BMI estimates over time. Just as importantly, this limitation issues a cautionary statement to researchers that the uncritical application of standard BMI correction procedures may fail to yield estimates that are unbiased approximations of clinical measures. Another important limitation of our study is that it divides race/ethnicity into two rather broad groups (Black and non-Black). While we believe that this is sufficient for our purposes, other studies may benefit from the development of separate corrections for other racial/ethnic groups, such as Hispanics. A third limitation of our correction procedure is that it is only directly applicable to NHIS data. But despite these limitations, our analyses have provided a set of statistical techniques that correct biases associated with proxy-reporting, and could be expanded further to adjust for the measurement error associated with self-reported BMI in the NHIS. Furthermore, we believe that the ideas developed here could be used to help minimize biases in other sources of epidemiologic data that use proxy-reports of height and weight to estimate BMI.

## Conclusion

It is imperative that researchers who measure BMI through reported estimates of height and weight think carefully about flaws in their data and how existing correction procedures might fail to account for them. The development of our correction procedure, which minimized the systematic underestimation of BMI due to the inclusion of proxy-reports of height and weight in the NHIS prior to 1997, represents an important step toward improving the quality of BMI estimates in a widely used source of epidemiologic data. As we have shown, however, correcting the downward bias in proxy-reports is only an initial step toward reducing the measurement error associated with BMI estimates in the NHIS or other data sources that rely on reports rather than direct measures of height and weight. Statistical adjustments that simultaneously account for period trends, demographic characteristics, and the interactions between them should be developed to improve the validity of reported estimates of BMI. Through the careful development of appropriate adjustment procedures for proxy- and self-reported data, epidemiologists will improve their capacity to document historic increases in body mass, despite changing data collection procedures over time. Through our detailed investigation of biases introduced into NHIS data by proxy-reporting, we hope to increase general awareness of these measurement issues and provide researchers with useful ideas for correcting patterns of BMI misreporting in other sources of data.

## Competing interests

The authors declare that they have no competing interests.

## Authors' contributions

ER devised the correction procedure for proxy-reported weight, acquired and analyzed the data used in this study, and wrote the initial draft of this manuscript. RU reviewed and helped simplify the correction procedure, revised the paper for intellectual content, and made substantial original contributions to the final draft. Both authors read and approved this manuscript.
